# 

**Published:** 1905-06

**Authors:** 


### CONTENTS.

ORIGINAL COMMUNICATIONS—

Is Scarlet Fever a Septicemia? By L. B. Clark, M.D. .......... 145
Elongation of the Cervix Prolapsus and Procidenture Vaginae et Uteri Principles and Operative Treatment. By A. Belcham Keyes, M.D. .......... 150
A Clinical Lecture. By William Seaman Bainbridge, A.M., M.D. .......... 159
Remarks of Dr. A. A. McKittrick at the American Anti-Tuberculosis League, Recently Convened in Atlanta, Ga. .......... 16

EDITORIAL NOTES AND COMMENTS—

Buttermilk .......... 169

The Natural History of Cancer .......... 171

CONTENTS CONTINUED .......... x

